# Heterologous expression of the *Monilinia fructicola CYP51* (*MfCYP51*) gene in *Pichia pastoris* confirms the mode of action of the novel fungicide, SYP-Z048

**DOI:** 10.3389/fmicb.2015.00457

**Published:** 2015-05-19

**Authors:** Fengping Chen, Dong Lin, Jingyuan Wang, Botao Li, Hongxia Duan, Junli Liu, Xili Liu

**Affiliations:** ^1^Key Laboratory of Plant Virology of Fujian Province, Institute of Plant Virology, Fujian Agriculture and Forestry UniversityFuzhou, China; ^2^Department of Plant Pathology, China Agricultural UniversityBeijing, China; ^3^Taizhou Entry-Exit Inspection and Quarantine BureauZhejiang, China; ^4^Department of Chemistry, College of Science, China Agricultural UniversityBeijing, China; ^5^State Key Laboratory of the Discovery and Development of Novel Pesticide, China Shenyang Research Institute of Chemical IndustryShenyang, China

**Keywords:** SYP-Z048, mode of action, heterologous expression, point mutation, *Monilinia fructicola*, Y136F

## Abstract

The novel agricultural fungicide 3-[5-(4-chlorophenyl)-2,3-dimethyl-3-isoxazolidinyl] pyridine (SYP-Z048) developed by China Shenyang Research Institute of Chemical Industry has been confirmed to be an ergosterol biosynthesis inhibitor (EBI). Previous studies have shown that EBIs target the proteins from a range of genes, including *CYP51*, *ERG2* and/or *ERG24*, and *ERG27*, which are involved in the ergosterol biosynthesis pathway. In the current study the *ERG2*, *ERG24*, and *ERG27* genes were cloned from wild type and resistant mutants of *Monilinia fructicola* in an attempt to clarify the target site of SYP-Z048. Comparative analysis of the deduced aa sequence of these genes, as well as *CYP51*, revealed several point mutations that resulted in amino acid variation among the sensitive and resistant isolates. However, sensitivity assays indicated that only one, the substitution of phenylalanine (F) for the tyrosine (Y) at 136 in *CYP51*, was correlated with reduced sensitivity to SYP-Z048. Heterologous expression of *MfCYP51*-*136Y* (*MfCYP136Y*) and *MfCYP51*-*136F* (*MfCYP136F)* in *Pichia pastoris* revealed that *MfCYP136F* significantly reduced sensitivity to SYP-Z048, increasing the average EC_50_ of the transformants 11-fold relative to those carrying *MfCYP136Y*. However, neither the additional copy of *MfCYP136Y* nor multiple copies of *MfCYP136F* were found to reduce sensitivity relative to the empty vector control or single copy transformants, respectively. Molecular docking experiments using SYP-Z048 with *HsCYP145Y* and the mutated version *HsCYP145F* as substitutes for *MfCYP136Y* and *MfCYP136F*, respectively, indicated that the reduced affinity of *HsCYP145F* for SYP-Z048 resulted from the loss of a hydrogen bond between the fungicide and the active site. Taken together these results indicate that *MfCYP51* is the major target site of SYP-Z048 in *M. fructicola*, which has important implications for the resistance management of this fungicide in the field.

## Introduction

Fungal diseases are one of the most important limiting factors in crop production, not only reducing yields and quality, but in some cases tainting the produce with toxins that are harmful to both livestock and human consumers. Although the development of plant cultivars exhibiting disease resistance is the method of choice for reducing the damage caused by pathogenic fungi, in practice many diseases require the application of agrochemicals for effective control, which has become a critical element in plant disease management programs.

The novel isoxazolidine class fungicide SYP-Z048 (Liu et al., 2012), 3-[5-(4-chlorophenyl)-2,3-dimethyl-3-isoxazolidinyl] pyridine (Supplementary Figure S1, Chen et al., 2014), developed by the China Shenyang Research Institute of Chemical Industry in 1997, has shown great promise for the control of a broad range of fungi and has been registered for the control of gray mold (*Botrytis cinerea*) on tomato in China under the trade name Junsiqi (25% EC, China Shenyang Research Institute of Chemical Industry, Shenyang, China) (Si et al., 2004). It has also been found that SYP-Z048 provides highly effective control of the closely related species *Monilinia fructicola* (G. Winter) Honey, a ubiquitous pathogen that is the primary causal agent of brown rot in stone fruit (EPPO/CABI^1^). The EC_50_ value for SYP-Z048 in baseline populations of *M. fructicola* is 0.017 μg/ml (Chen et al., 2012), which is similar to the values for propiconazole (0.03 μg/ml) (Zehr et al., 1999) and tebuconazole (0.016 μg/ml) (Yoshimura et al., 2004), and field trials have indicated that SYP-Z048 effectively controls brown rot in peach orchards (Chen et al., 2014).

Biochemical analysis has shown that SYP-Z048 inhibits ergosterol biosynthesis in *B. cinerea* (Han et al., 2006). Ergosterol biosynthesis inhibitors (EBIs) have been subcategorized further according to their target sites within the ergosterol biosynthesis pathway: inhibitors of 14α demethylation (known as DMIs), inhibitors of sterol Δ14 reduction and/or Δ8 → Δ 7-isomerisation, and inhibitors of C-4 demethylation (Siegel, 1981; Leroux et al., 2002). The target proteins of these groups are the C-14 sterol demethylase, C-8 sterol isomerase and/or C-14 sterol reductase, and 3-keto-steroid reductase, respectively, which are encoded by the genes *CYP51, ERG2* and/or *ERG24*, and *ERG27*. A preliminary study investigating the cross resistance of SYP-Z048 with propiconazole and amino acid (aa) changes in *MfCYP51* has indicated that SYP-Z048 is likely to be a DMI (Chen et al., 2012). However further investigation is required to confirm these initial results and to determine whether the other three enzymes could also be target sites for SYP-Z048.

The objective of the current study was to clarify the mode of action of SYP-Z048 using comparative sequence analysis of the EBI target genes from wild-type and resistant mutants of *M. fructicola*, as well as by the heterologous expression of candidate resistance genes in combination with sensitivity assays. The methylotrophic yeast *Pichia pastoris*, which is commonly used for heterologous gene expression in both research and industrial production (Cos et al., 2006; Bollok et al., 2009), was selected for the study because as a eukaryote, *P. pastoris* has many advantages over bacterial expressions systems and as a fungus also exhibits sensitivity to EBI fungicides.

## Materials and methods

### Isolates

Eight single-spore *M. fructicola* isolates exhibiting different EC_50_ values for SYP-Z048 (Chen et al., 2012) were selected for the study, including 3 sensitive isolates MSB11, MPA18 and MFJ2 with EC_50_ values of 0.011, 0.013 and 0.033 μg/ml, respectively; 4 highly resistant isolates B5013, B6012, B506 and B511 with EC_50_ values of 0.342, 0.570, 0.820 and 0.886μg/ml, respectively; and one isolate exhibiting low resistance A3081 with EC_50_ values of 0.097 μg/ml. Resistant isolates of B5013, B6012, B506, and B511 were generated via ultraviolet irradiation of conidia on SYP-Z048-amended media, while A3081 was created via ultraviolet irradiation of mycelium. All of the *M. fructicola* isolates were retrieved from filter paper storage and cultured as described previously (Chen et al., 2012). The *Pichia pastoris* isolate, GS115, was used for the heterologous expression in conjunction with the pPIC9K vector, which were kindly donated by Dr. Xiuguo Zhang from the Shandong Agricultural University.

### Cloning of the *ERG2*, *ERG24*, and *ERG27* genes from *M. fructicola*

Genomic DNA was extracted from the *M. fructicola* isolates using the Cetyl Trimethylammonium Bromide (CTAB) method from a previous study (Chen et al., 2012) with slight modifications. The mycelia were collected from solid cultures grown on YGA medium (0.5% yeast extract, 1.8% glucose, and 1.2% agar) and snap-frozen in liquid nitrogen before being ground with a pestle and mortar in liquid nitrogen. The powdered samples (0.1 g) were transferred to centrifuge tubes containing 750 μl extraction buffer (2% CTAB, 100 mM Tris-HCl pH 8.0, 20 mM EDTA pH 8.0, 1.4 M NaCl) and 2 μl RNase A (100 mg/ml, Qiagen Inc., Valencia, CA). After incubation for 1.5 h at 65°C with occasional mixing, the protein was removed by the addition of one volume of phenol-chloroform-isoamyl alcohol (25:24:1) and centrifugation at 12,000 g for 10 min, before the DNA was precipitated from the supernatant with one volume of isopropyl alcohol for 10 min at room temperature (23°C). The suspension was centrifuged at 12,000 g for 10 min and the pellet washed with 75% ethanol. The resulting DNA was dried in a laminar flow hood before being resuspended in TE buffer (10 mM Tris-HCl and 1 mM EDTA, pH 8.0).

Fragments of the *ERG2, ERG24* and *ERG27* genes were initially amplified from isolate MSB11 using the following primer sets: erg2F1/erg2R1, erg24F1/erg24R1 and erg27F1/erg27R1, respectively, which were designed to the sequences of the homologous genes in the closely related species *B. cinerea* and *S. sclerotiorum* (Supplementary Table S1). The PCR was performed using 50 μl reaction mixes containing 1× PCR buffer, 50 ng of DNA, 0.4 μM each primer, 25 μM each dNTP, and 1 U of Taq DNA polymerase (TransGen Biotech Co., Beijing) and processed in a MyCycler thermal cycler (Bio-Rad Laboratories, Hercules, CA) with the following program: 4 min at 95°C; 32 cycles of 30 s at 95°C, 30 s at 60°C, and 2 min at 72°C; followed by a final extension step of 10 min at 72°C. A slightly lower annealing temperature of 57°C was used in the case of *ERG2*. The PCR products were separated by electrophoresis using 1.0% agarose gels in 1× Tris-acetate-EDTA buffer (40 mM Tris acetate and 1 mM EDTA, pH 8.0), and purified using a gel extraction kit (TransGen Biotech) before being ligated into the pEasy T3 vector (TransGen Biotech) and commercially sequenced (Sunbiotech Co., Beijing) using the vector specific primers M13F and M13R. The resulting DNA sequences were analyzed using the DNAMAN5.2.2.0 software package (Lynnon BioSoft, Quebec, Canada).

The flanking sequences of the *ERG2, ERG24*, and *ERG27* fragments were amplified by site-finding PCR (Tan et al., 2005). Specific primers were designed to each of the gene fragments to amplify both the upstream (-UR1, -UR2 and -UR3) and downstream (-DF1, -DF2 and -DF3) sequences (Supplementary Table S1). The universal (siteFinder1 and siteFinder2), and specific primers (SFP1 and SFP2) used have been described previously (Tan et al., 2005) (Supplementary Table S1). Details of the site finding and nested PCR (both primary and secondary) are shown in Supplementary Table S2. The resulting PCR products were separated and sequenced as described above and the contiguous sequences combined to obtain the complete sequence of *ERG2, ERG24* and *ERG27* using the DNAMAN5.2.2.0 software package. The open reading frames for the three genes were inferred from similarities with homologous sequences from the closely related species *B. cinerea* and *S. sclerotiorum*, which were retrieved from the Broad Institute of MIT and Harvard database (http://www.broadinstitute.org//scientific-community/data): Accession numbers BC1G_03303.1, BC1G_00806.1, and BC1G_14884.1, for *ERG2, ERG24* and *ERG27*, respectively in *B. cinerea*, and SS1G_06738.3, SS1G_00326.3 and SS1G_00326.3 in *S. sclerotiorum*. Introns were confirmed by amplifying the complete *ERG2, ERG24* and *ERG27* sequences using cDNA from isolate MSB11 as a template.

The complete gene sequences for *ERG2*, *ERG24* and *ERG27* were then amplified from the other 7 isolates using PCR and the following primer sets erg2F2/erg2R2, erg24F2/erg24R2 and erg27F2/erg27R2, respectively (Supplementary Table S1)., which were designed base on the sequences from MSB11. The conditions for the PCR were identical to those used to amplify the gene fragments from MSB11, with the exception of the higher annealing temperatures used: 66°C, 64°C and 58°C for *ERG*2, *ERG24* and *ERG27*, respectively. The resulting PCR products were then cloned and sequenced as described above. In addition, the complete *ERG24* and *ERG27* gene sequences were also sequenced using the primers erg24R1 and erg27R1, respectively.

### RNA isolation, cDNA synthesis and amplification of the *MFCYP51* gene in *M. fructicola* isolates

The *MfCYP51* gene was amplified from cDNA libraries of both the susceptible and resistant isolates MSB11 and B511, respectively. The mycelium was prepared as described above and the total RNA extracted using the SV Total RNA Isolation System (Promega Corp., Madison, WI), following the protocol of the manufacturer. The absence of genomic DNA contamination was verified by PCR using the purified RNA samples as a template. The cDNA was then synthesized using the iScript cDNA Synthesis Kit (Bio-Rad). The forward primer CYP51Fc was designed to contain an *EcoR*I restriction site (in italic) and His-tag (underlined) at the 5′ terminal; 5′-(G*GAATT*CCACCACCACCACCACCACATGGGTGTTCTCGAGACCAT)-3′, while the reverse primer CYP51Rc contained a *Not*I restriction site (in italic) at the 5′ terminal; 5′-(ATAAGAAT*GCGGCCGC*TTATCGTCTCTCCCATGCCA)-3′. The PCR was performed in 50 μl reaction mixtures containing 1× Prime STAR buffer, 15 ng of cDNA, 0.2 μM of each primer, 25 μM of each dNTP, and 1.25U of PrimeSTAR HS DNA Polymerase (Takara, Inc., Dalian, China), and amplified using 35 cycles of 98°C for 10 s and 68°C for 2 min. The resulting PCR products were cloned and sequenced as described above.

### Construction of pPIC9K-MfCYP51 expression vectors, transformation and screening

The vector construction and transformation in *P. pastoris* were carried out according to the protocol of the pPIC9K manufacturer (Invitrogen Co., Carlsbad, CA, USA). The pPIC9K-MfCYP51 expression vector was constructed by inserting the PCR products into the pPIC9K vector as an *EcoR*I/*No*tI (Thermo Fisher Scientific (China) Co., Ltd. Beijing) restriction fragment. The PCR products and vectors were double digested at 37°C for 16 h, before being purified and ligated using T4 DNA ligase (TransGen Biotech Co., Beijing). The plasmids were then transformed into *Escherichia coli* competent cell Trans1-T1 (TransGen Biotech Co., Beijing), and the positive colonies selected using PCR with the universal primer set 5′AOX and 3′AOX (Supplementary Table S1). The presence of the insert was confirmed by sequencing. The two pPIC9K-MfCYP51 expression vectors were designated pPIC-CYP136Y and pPIC-CYP136F corresponding to the amino acid found at position 136 in the *MfCYP51* genes of the susceptible and resistant isolates MSB11 and B511, respectively.

The plasmids pPIC-CYP136F, pPIC-CYP136Y and the empty vector pPIC9K were linearized by *Sal*I (Bio-Rad, USA) digestion and transformed into *P. pastoris* GS115 competent cells using 2 mm cuvettes and a MicroPulser (Bio-Rad, USA) set at 1.5 kV. Immediately after electroporation 1 ml cold sorbitol (1 M) was added to the cuvette before the cells were transferred to 5 ml-sterilized tubes and incubated at 30°C without shaking for 1 h. The cells were then plated on minimal dextrose media (1.34% yeast nitrogen base, 0.00004% biotin, 2% dextrose, 1.8% agar) and incubated at 30°C for 1 h. The resulting colonies were then screened on minimal methanol media (1.34% yeast nitrogen base, 0.00004% biotin, 0.5% methanol, 1.8% agar), and then on YPD media (1% yeast extract, 2% peptone, 2% dextrose, and 2% agar) containing 250, 500, 750, and 1000 μg/ml G418. After 2–3 days of incubation at 30°C, the transgene status of the transformants was confirmed by PCR amplification of the *MfCYP51* or AOX gene from genomic DNA using the CYP51F2/CYP51R2 and 5′AOX/3′AOX primer sets, respectively (Supplementary Table S1). The genomic DNA of the transformants was extracted using the Yeast DNA Extraction Kit (Tiangen Biotech CO. LTD, Beijing), according to the protocol of the manufacture and the PCR conducted as described for *ERG2* above, but with an annealing temperature of 55°C.

### Heterologous expression of the *MfCyp51* gene in *P. pastoris*

The expression of *MfCYP51* in the *P. pastoris* transformants was confirmed using RT-PCR. Total RNA was extracted from four transformants of both GS115-pPIC-CYP136F and GS115-pPIC-CYP136Y, in addition to one transformant of GS115-pPIC9K as well as the parental isolate GS115, using the SV Total RNA Isolation System (Promega Corp., Madison, WI), according to the protocol of the manufacture. The cDNA synthesis and RT-PCR was conducted using the same procedure used for *M. fructicola*, described above.

### Sensitivity of transformants and parental isolate GS115 to SYP-z048

The SYP-Z048 sensitivity of both the transformants and parental isolate GS115 was determined using a modified method based on the protocol of a previous study (CLSI, 2008). Briefly, each isolate was incubated in 20 ml YPD liquid medium at 28°C, with shaking (200 rpm) until the OD_600_ of the cultures reached 1.1–1.3. Next 10 μl of the cultures were transferred to 200 ml fresh YPD containing varying concentrations of SYP-Z048 (0, 0.125, 0.25, 0.5, 1, and 2 μg/ml), and incubated until the OD_600_ of the non-fungicide control was around 2.0. At this point the OD_600_ of the other isolates was measured and used to calculate their EC_50_ values. The experiment was conducted 3 times in total and the significance levels for mean EC_50_ values determined using the GLM procedure with LSD comparisons at *p* = 0.05 (SAS software ver. 8.0).

### Southern blot analysis of *MfCyp51* in GS115-pPIC-CYP136F and GS115-pPIC-CYP136Y transformants

Southern blot analysis was conducted to evaluate the influence of *MfCYP51* copy number on the SYP-Z048 sensitivity of the transformants. Genomic DNA was extracted from the eight transformants according to protocol of the yeast supplier (Invitrogen Co., Carlsbad, CA, USA). Approximately 8 μg of genomic DNA was digested with *EcoR* I, separated on a 2.0% agarose gel and transferred to a Hybond–N+ positively charged nylon membrane (GE Healthcare, Pittsburgh, PA). The 471 bp probe was amplified from isolate MSB11 using the cypF5/cypR primer set (5′-GAAACTCTCCGTCTCCACAC-3′ and 5′-TCGTCTCTCCCATGCCACAA-3′), and the subsequent labeling of the probe, hybridization and detection were all performed using the MyLab DIG Labeling and Hybridization Detection System-DIG DNA PCR Labeling Kit (MyLab Corp., Beijing) according to the protocol of the manufacturer.

### Computer modeling of SYP-z048 molecular docking

The molecular docking of SYP-Z048 in the *CYP51* binding pocket was simulated using the crystal structure of the *HsCYP51* protein from *Homo sapiens* bound to econazole (ECN) within the 3JUS complex, which was retrieved from the Protein Data Bank (PDB code : 3JUS). The experiments were conducted using the Sybyl 7.3 software package. The substitution of phenylalanine for the tyrosine at position 145 of *HsCYP51*, which corresponds to Y136F in *MfCYP51* of *M. fructicola* (Lepesheva et al., 2003), was conducted using the Biopolymer-Mutate Monomers module, while the energy minimization of the new complex was calculated using the Minimize Subset module. Residues in contact with the mutation within a 10Å radius were optimized. The Energy minimization was performed using the Tripos force field with a convergence criterion of 0.005 kcal/mol/Å. Charges were calculated by the Gasteiger-Marsili method. The simulation was performed with 10000 cycles and the lowest energy conformations selected. The 3D conformations of SYP-Z048 were built according to the structure of econazole within the 3JUS complex and geometrically optimized using the Build/Edit module, while the docking experiments were carried out using the Surflex-Dock algorithm module. Complex X-ray structures were generated when SYP-Z048 was docked in the active site of both the F145 and 145Y forms of *HsCYP51*. The best ligand pose was then selected based on the top Surflex-Dock energy score. The affinity between the mutation site and SYP-Z048 were analyzed based on the energy score and binding mode of the active site.

## Results

### Isolation of the *ERG2, ERG24*, and *ERG27* sequences from *M. fructicola*

The complete nucleotide sequences of *ERG2*, *ERG24* and *ERG27*, which encode a C-8 isomerase, C-14 sterol reductase and 3-keto-steroid reductase, respectively, were successfully cloned from the SYP-Z048-sensitive isolate MSB11. The genes were 779, 1609 and 1704 bp in length and contained 2, 2, and 1 introns, respectively. The intron splicing sites were verified by PCR using cDNA as a template, and the deduced amino acid (aa) sequences were found to be 215, 496 and 541 in length (Table 1). The gene sequences of *ERG2*, *ERG24* and *ERG27* were submitted to the NCBI database under the following accession numbers: KP144211, KP144211 and KP144211, respectively.

**Table 1:** **Characteristics of the *M. fructicola* genes, *ERG2*, *ERG24*, and *ERG27* which are the target genes of ergosterol biosynthesis inhibitors besides *CYP51***.

**Gene**	**Protein**	**Size (bp)**	**No. of introns**	**No. of aa**	**Accession number**
*ERG2*	C-8 sterol isomerase	779	2	215	KP144211
*ERG24*	C-14 sterol reductase	1609	2	496	KP144212
*ERG27*	3-keto-steroid reductase	1704	1	541	KP144213

The results of a BLAST analysis showed that the deduced aa sequences of *ERG2*, *ERG24* and *ERG27* from *M. fructicola* exhibited a high degree of similarity to the corresponding sequences from closely related species. The deduced aa sequence of *ERG2* exhibited 94% sequence identity to those of *Sclerotinia sclerotiorum* (Genbank accession number XP_001592497.1), *Sclerotinia borealis* (ESZ97016.1), *Botryotinia fuckeliana* (XP_001558454.1); 83–84% to those of *Glarea lozoyensis* (XP_008081182.1) and *Marssonina brunnea* (XP_007297759.1); and 75–78% to those of *Blumeria graminis* f. sp. *tritici* (EPQ63078.1), *B. graminis* f. sp. *hordei* (CCU82464.1), *Aspergillus terreus* (XP_001212934.1) and *Byssochlamys spectabilis* (GAD92783.1). The deduced aa sequence of *ERG24* exhibited 94–96% to those of *B. fuckeliana* (XP_001546819.1), *S. sclerotiorum* (XP_001591653.1) and *S. borealis* (ESZ98063.1); and 70–78% to those of *M. brunnea* (XP_007290035.1), *G. lozoyensis* (XP_008080468.1), *B. graminis* f. sp. *tritici* (EPQ63705.1) and *B. graminis* f. sp. *hordei* (CCU75655.1). The deduced aa sequence of *ERG27* exhibited 87% sequence identity to those of *S. sclerotiorum* (XP_001598240.1) and *S. borealis* (ESZ94691.1); 82–85% to those of *B. fuckeliana* (AAO64345.1) and *Botrytis pseudocinerea* (AFA52824.1); and 58–61% to those of *M. brunnea* (XP_007288930.1) and *G. lozoyensis* (XP_008079142.1).

### Sequence variation between the *ERG2, ERG24, ERG27* and *MfCyp51* genes of SYP-z048-sensitive and -resistant isolates of *M. fructicola*

The sequences of *MfCYP51* genes were retrived from previous study(Chen et al., 2012), which found **one** aa alterations corresponding to the substitution of tyrosine (Y) for the phenylalanine (F) at 136 was correlated with SYP-Z048 resistance. The *ERG2*, *ERG24*, and *ERG27* genes from three sensitive wild-type isolates as well as the five SYP-Z048-resistant isolates were compared to identify any mutations that might be associated with SYP-Z048 resistance as well. Analysis of the DNA sequence found several nucleotide variations in *ERG24* and *ERG27* gene (Supplementary Table S3), and the deduced aa sequences comparisons revealed that three and one deduced aa variations occurred among three sensitive isolates in *ERG24* and *ERG27*, respectively. AA sequences of all 5 resistant isolates were identical to sensitive isolate of MSB11 in *ERG24* and *ERG27*. None of the isolates exhibited any changes in the deduced aa sequences of their *ERG2* genes. (Table 2).

**Table 2:** **Amino acid variations associated with the *ERG2*, *ERG24*, and *ERG27* genes of sensitive isolates and SYP-Z048-resistant mutants of *M. fructicola***.

**Isolate**	Phenotype^*^	**Variation at amino acid position**				
		***ERG2***	***ERG24***			***ERG27***
			**186**	**193**	**327**	**263**
MSB11	S	Identical for all isolates	T	V	G	V
MPA18	S		P	A	S	A
MFJ2	S		P	A	S	A
A3081	LR		T	V	G	V
B5013	HR		T	V	G	V
B6012	HR		T	V	G	V
B506	HR		T	V	G	V
B511	HR		T	V	G	V

* *S, LR, and HR indicated that the isolates was sensitive, low resistant and highly resistant to SYP-Z048*.

### Heterologous expression of *MfCyp51* genes in *P. pastoris* and its effect on SYP-z048 sensitivity

**Figure 1:**
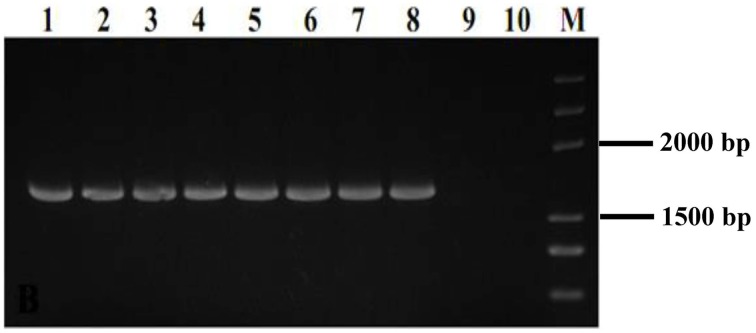
**PCR analysis used to confirm the presence of *MfCYP51* in 8 *Pichia pastoris* transformants**. Lanes 1–4, Transformants carrying the vector pPIC-CYP136F containing *MfCYP51* with phenylalanine at position of 136; Lanes 5–8, Transformants carrying the vector pPIC-CYP136Y containing *MfCYP51* with tyrosine at position of 136; Lane 9, Transformant carrying the empty vector pPIC9K, which did not contain *MfCYP51*; Lane 10, Untransformed parental isolate GS115; M, DNA marker.

The two *MfCYP51* variants, *MfCYP136Y*, and *MfCYP136F*, were selected for heterologous expression in *P. pastoris* to validate whether the observed SYP-Z048 resistance in *M. fructicola* did indeed result from the Y136F mutation. The presence of the pPIC-CYP136Y, pPIC-CYP136F, and pPIC9K vectors in the *P. pastoris* transformants was confirmed by PCR analysis (data not shown), while RT-PCR verified that *MfCYP51* was expressed in all eight transformants carrying *MfCYP136Y* or *MfCYP136F*, but not in those carrying the pPIC9K “empty” vector or in the parental isolate, GS115 (Figure 1).

**Figure 2:**
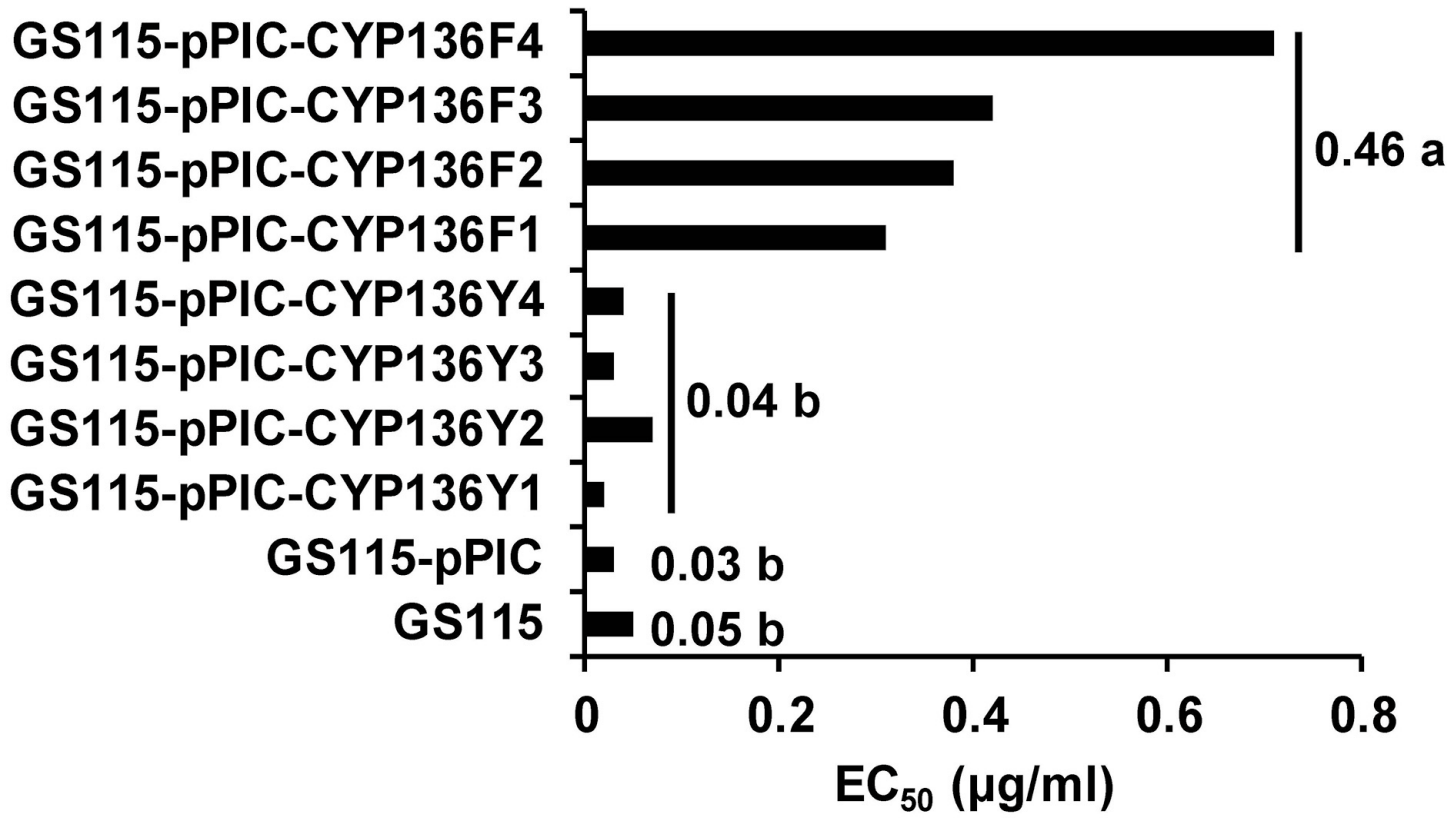
**Sensitivity of 8 *Pichia pastoris* transformants expressing either *MfCYP136F* (GS115-pPIC-CYP136F1-4) or *MfCYP136Y* (GS115-pPIC-CYP136Y1-4) to the EBI fungicide SYP-Z048, compared to the parental isolate (GS115) and a transformant carrying the empty vector (GS115-pPIC), which were used as controls**. Bars indicate the mean EC_50_ of the two different transformant populations. Values followed by different letters are significantly different at *p* = 0.05.

Expression of *MfCYP136F* resulted in significantly reduced sensitivity to SYP-Z048, with the mean EC_50_ for transformants carrying *MfCYP136F* being 11 times higher than for those carrying *MfCYP136Y* (Figure 2), which did not significantly differ from the transformant carrying the empty vector or the parental isolate, GS115.

### Influence of *MfCyp51* copy number on sensitivity to SYP-z048

**Figure 3:**
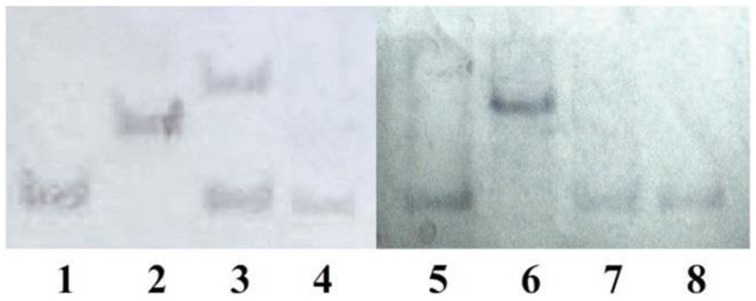
**Southern blot analysis of 8 *Pichia pastoris* transformants using a fragment of *MfCYP51* as the probe**. Lanes 1–4, Transformants carrying *MfCYP136F* (GS115-pPIC-CYP136F1-4); Lanes 5–8, Transformants carrying *MfCYP136Y* (GS115-pPIC-CYP136Y1-4). Although, transformant GS115-pPIC-CYP136F3 (lane 3) can be seen to contains two copies of *MfCYP136F*, sensitivity tests indicated that its EC_50_ was not significantly different to those of the transformants carrying a single copy (lanes 1, 2, and 4).

Southern blot analysis of the transformants carrying *MfCYP136F* or *MfCYP136Y* indicated that only one of the transformants, GS115-pPIC-CYP136F3, carried more than one copy of the *MfCYP51* gene (Figure 3). However, although this transformant carried two copies of the mutated version of the *MfCYP136F* gene, which is associated with SYP-Z048 resistance, its EC_50_ was not significantly different to the transformants carrying a single copy (Figure 2).

### Affinity of the mutated *MfCyp136F* for SYP-z048

**Figure 4:**
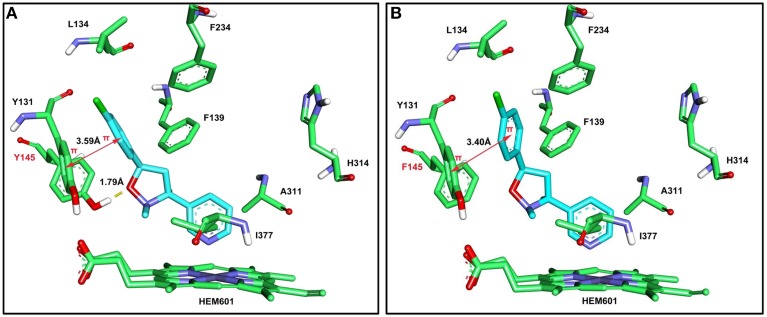
**Molecular docking interactions between the *HsCYP51* from *Homo sapiens* and SYP-Z048 (in blue)**. A wild-type (Y145) and mutated (145F) version of *HsCYP51* were assessed, which corresponded to the Y136F mutation in *MfCYP51* of *Monilinia fructicola*. **(A)** 3D representation of the interactions between SYP-Z048 and the amino acid residues of the wild-type binding pocket, which results in the formation of hydrogen bond (yellow dash) and π −π stacking (magenta arrow). **(B)** The interactions between SYP-Z048 and the amino acid residues of the Y145F mutated protein, which results in the loss of the hydrogen bond.

The computer models produced in the molecular docking analysis indicated that the Y145F mutation in *HsCYP51*, which corresponds to Y136F in *M. fructicola* (Lepesheva et al., 2003), changed the conformation of the binding pocket and reduced its potential to bind SYP-Z048. Although the π −π stacking was little changed (Figure 4), the loss of the hydrogen bond between *CYP51* and SYP-Z048, as a result of the substitution of phenylalanine for the tyrosine at position 145, led to altered affinity scores of 6.73 and 5.49 for the wild-type and mutated *CYP51*, respectively.

## Discussion

The development of resistance to fungicides often involves modifications to the biochemical target site that decreases its affinity for the fungicide. An understanding of a fungicide's mode of action can therefore be helpful when investigating the molecular basis of resistance in plant pathogens, and similarly, analysis of resistant mutants can provide clues to the mode of action of fungicides. The combination of genetic techniques and biochemical analysis is therefore an extremely useful approach to determining the mode of action of new fungicides. For example, this approach successful identified *PiCesA3* as the target site for the fungicide mandipropamid in the oomycete pathogen *Phytophthora infestans* (Blum et al., 2010).

Previous research has shown that SYP-Z048 acts as an EBI (Han et al., 2006). The target sites of agricultural EBIs include *ERG2*, *ERG24*, *ERG27* and *MfCYP51*, which encode a C-8 sterol isomerase, C-14 sterol reductase, 3-keto-steroid reductase and C-14 sterol demethylase, respectively, all key enzymes in the ergosterol biosynthesis pathway. Indeed, the importance of these target proteins has been used to differentiate EBI fungicides (Siegel, 1981; Leroux et al., 2002). In our previous study several highly resistant mutants of *M. fructicola* were produced by UV-irradiation of spores (Chen et al., 2012), where only the *MfCYP51* gene in sensitive and resistant isolates was analyzed. In the current study the *ERG2*, *ERG24* and *ERG27* genes were cloned and sequenced to establish whether these genes were targeted by SYP-Z048 and whether mutations in these genes contributed to fungicide resistance. Those three genes were firstly cloned in *M. fructicola* isolates. The deduced aa sequences of the *ERG2* and *ERG24* exhibited high similarity to sequences from the closely related species *S. sclerotiorum*, *S. borealis*, and *B. fuckeliana*, sharing 94% or greater sequence identity, while the sequence identity of *ERG27* ranged from 82 to 87%. These results confirmed that the genes isolated from *M. fructicola* were indeed homologs of *ERG2, ERG24* and *ERG27*. Although the sequences of *ERG2*, *ERG24*, *ERG27* and *MfCYP51* were found to contain several point mutation that resulted in aa alterations, only one mutation, corresponding to a substitution of phenylalanine (F) for the tyrosine (Y) at 136 in *MfCYP51* was found to be correlated with resistance in fungicide sensitivity tests.

Heterologous expression of the wild type and mutated *MfCYP51* genes in *P. pastoris* confirmed the Y136F mutation was associated with resistance, and on average reduced sensitivity to SYP-Z048 11-fold. However, comparison of the difference between the mean EC_50_ values for the resistant *M. fructicola* mutants and *P. pastoris* transformants relative to their base line sensitivities, 32.7 ± 12.4 and 11.4 ± 4.4, respectively, indicated that the Y136F mutation was less effective in *P. pastoris*. A possible explanation for this observation might be that the *M. fructicola* mutants had additional non-target site mechanisms for resistance, which is supported by the observation that the sensitivity of the *M. fructicola* mutants to SYP-Z048 varied greatly, ranging from 20-fold to 52-fold compared to the baseline sensitivity. An alternative explanation might be that the cell membranes of *P. pastoris* could have a different permeability to SYP-Z048 than those of *M. fructicola*.

The mechanism by which the Y136F mutation reduces sensitivity to SYP-Z048 appears to be associated with an alteration to the structure of the *MfCYP51* protein. It has previously been shown that the conserved residues of *CYP51* are most likely to have a structure role. For example, Y116 in *Trypanosoma brucei*, which corresponds to Y136 in *M. fructicola* is an essential residue involved in forming the surface of *CYP51* substrate binding cavity and provides heme support (Lepesheva et al., 2003; Lepesheva and Waterman, 2011). The replacement of such crucial residues is known to change the efficacy of substrate binding and catalysis of enzymes (Lepesheva et al., 2003, 2007). The 3 dimensional modeling conducted in the current study supported this hypothesis, as the capacity for *MfCYP51* to bind SYP-Z048 was reduced when the tyrosine at position 136 was replaced by phenylalanine, which resulted in the loss of a hydrogen bond between the substrate and the active site.

Previous studies have shown that, in general, increased numbers of target genes reduce sensitivity to fungicides. For example, the expression of an additional *B*. *graminis CYP51* gene in *B*. *cinerea* was found to reduce sensitivity to triadimefon (Yan et al., 2012). Furthermore, it has been reported that multi-copies of *CYP51* can reduce sensitivity to DMI fungicides in several species of fungi including *Candida glabrata* (Marichal et al., 1997). *C*. *albicans* (Selmecki et al., 2006) and *Cryptococcus neoformans* (Sionov et al., 2010). However, the current study found no evidence of this phenomenon in *P. pastoris*, with neither the additional copy of *MfCYP136Y* nor the second copy of *MfCYP136F* reducing sensitivity relative to the empty vector control or single copy transformant, respectively. These results are consistent with a similar study in *Saccharomyces cerevisiae*, in which heterologous expression of *MfCYP51* failed to reduce sensitivity to several triazoles fungicides including propiconazole, fenbuconazole or tebuconazole (Schnabel and Dai, 2004), although it did reduced sensitivity to myclobutanil. The conflicting results indicate that the interactions of fungicides with *CYP51* are complex and could be influenced by several factors.

Attempts were made to transform isolates of *M. fructicola* so that the influence of *MfCYP136Y* could be studied directly. However, no stable transformants could be generated by site-directed mutagenesis of the sensitive isolate using agrobacterium T-DNA mediated transformation (Lee and Bostock, 2006). Perhaps it is not surprising that it was difficult to replace *CYP51* given that it occurs as a single copy and plays an essential role in cellular metabolism.

The combination of genetic analysis and fungicide sensitivity assays used in the current study provide strong evidence that *MfCYP51* is the usual target site of SYP-Z048 in *M. fructicola*. These results suggest that although SYP-Z048 has previously been classified as an isoxazolidine fungicide (Shaber et al., 2001; Liu et al., 2012), its mode of action indicates it shows greater similarity to pyridine fungicides. These results are important when considering fungicide resistance strategies for SYP-Z048, which is a highly effective fungicide for the control of *M. fructicola* (Chen et al., 2014). Given that incomplete cross-resistance has been reported among other DMI fungicides (Erickson and Wilcox, 1997; Mavroeidi and Shaw, 2005; Hawkins et al., 2014), it is likely that novel DMI fungicides such as SYP-Z048 could experience increased risk of resistance if fungal populations have already encountered similar selection pressures in the field.

## Author contributions

XL and FC designed the experiments. FC and JW performed all the experiments except for the 3D modeling. DL, BL, and HD performed the 3D modeling. FC, XL, HD, and JL. interpreted the results. FC and XL wrote the paper. All authors reviewed the manuscript.

### Conflict of interest statement

The authors declare that the research was conducted in the absence of any commercial or financial relationships that could be construed as a potential conflict of interest.

## Footnotes

^1^EPPO/CABI. Data sheets on quarantine pests: *Monilinia fructicola* (2010). Available from: https://www.eppo.int/QUARANTINE/fungi/Monilinia_fructicola/MONIFC_ds.pdf (Date of access: *Jan*/19/2015)
