# Supplementary material for: Heterologous expression of the Monilinia fructicola CYP51 (MfCYP51) gene in Pichia pastoris confirms the mode of action of the novel fungicide, SYP-Z048
Source: Front Microbiol. 2015 May 19;6:457. doi: 10.3389/fmicb.2015.00457 (PMC4437033; doi:10.3389/fmicb.2015.00457)
Supplement: Supplementary file 1 [file DataSheet1.ZIP › S-Tables/S1.PDF]

Supplementary table s1: Primers used in this study

Primer	Sequence (5’----3’)	Description	Source
CYPF5	GAAACTCTCCGTCTCCACAC	Amplification of hybridization probe	This study,
CYPR	TCGTCTCTCCCATGCCACAA		
CYPR5	GTGTGGAGACGGAGAGTTTC	Sequencing primer for *MfCYP51*	This study
ERG2F1	GGCTATGGGTGCTATGTAT	Amplification of *ERG2* fragment	This study
ERG2R1	ATCCAACCTCTAGCATACTC		
ERG24F1	CGAAATGACTCCTAAGAAG	Amplification of ERG24 fragment	This study
ERG24R1	CTAATAGATA CCTGGGATAA T		
ERG27F1	GATGCTACTACAATGGATGGTC	Amplification of ERG27 fragment	This study
ERG27R1	TACCTTCCCA TCCCCAACC		
ERG2F2	CCTGCCAGCTACCACCGACT	Amplification of complete *ERG2*	This study
ERG2R2	CAAAAGGGGCGTGCTGAGGAG		
ERG24F2	CCATCCTTCTACTGAGCGTTC	Amplification of complete *ERG24*	This study
ERG24R2	ACACTCTTGCCCACCTGTAA		
ERG27F2	GAGAACGATAGCCATATACAACT	Amplification of complete *ERG27*	This study
ERG27R2	ACACAAACATGATAGATCGCA		
ERG2-DF1	GAAGGACATACCGGTCGTCATAC	Downstream amplification of *ERG2*	This study
ERG2-DF2	CGGTACTCAACTTGCCTATACTCCAGG		
ERG2-DF3	GCAGTATAAGATGGATGCGGCTTG		
ERG2-UR1	CAAGCCGCATCCATCTTATACTGC	Upstream amplification of *ERG2*	This study
ERG2-UR2	CCTGGAGTATAGGCAAGTTGAGTACCG		
ERG2-UR3	GGAACTCCGCAACTCGCCCGA		
ERG24-DF1	CGGACTCATGTTATCATTCGGTGAC	Downstream amplification of *ERG24*	This study
ERG24-DF2	GGTGGTGTTCTTGCGGTCCTAGGCT		
ERG24-DF3	TCAGGACCGACCCCACAGATCCC		
ERG24-UR1	ATCGATTTCGCCGAGTAGTGGGAGAGT	Upstream amplification of *ERG24*	This study
ERG24-UR2	AATGACCACCAACAGCCAGCTCGC		
ERG24-UR3	CCAGGCTTGACGCTGAAGCTGCGAAT		
ERG27-DF1	CGGAGATCATGCCCTTGAACTTTGTC	Downstream amplification of *ERG27*	This study
ERG27-DF2	CCAGTATGGGTAGCATTATCCCCCG		
ERG27-DF3	GGGGATCGGCCACTGATTCGAGTG		
ERG27-UR1	CCTGACCATCCTCCAATACCTGCAT	Upstream amplification of *ERG27*	This study
ERG27-UR2	CCCCATCCCCAAGATCTCTGCTCGC		
ERG27-UR3	GCCCACCTGTCTTGTTGAACGTCTGG		
siteFinder1	CACGACACGCTACTCAACACACCACCTCGCA CAGCGTCCTCAAGCGGCCGCNNNNNNGCCT	Universal primers for SiteFinder PCR	(Tan et al. 2005)
siteFinder2	CACGACACGCTACTCAACACACCACCTCGCA CAGCGTCCTCAAGCGGCCGCNNNNNNGCGC		
SFP1	CACGACACGCTACTCAACAC	Specific primers for SiteFinder PCR	(Tan et al.
SFP2	ACTCAACACACCACCTCGCACAGC		

			2005)
CYP51Fc	GGAATTCCACCACCACCACCACCACATGGGTGTTCTCGAGACCAT	Cloning of *MfCYP51* for expression vector construction	This study
CYP51Rc	ATAAGAATGCGGCCGCTTATCGTCTCTCCCATGCCA		
5’AOX	GACTGGTTCCAATTGACAAGC	Verification of *MfCYP51* in transformants	Invitrogen Co.
3’AOX	GCAAATGGCATTCTGACATCC		
CYP51F2	ATGGGTGTTCTCGAGACCAT	Verification of *MfCYP51* in transformants	This study
CYP51R2	TTATCGTCTCTCCCATGCC		
