# Supplementary material for: Heterologous expression of the Monilinia fructicola CYP51 (MfCYP51) gene in Pichia pastoris confirms the mode of action of the novel fungicide, SYP-Z048
Source: Front Microbiol. 2015 May 19;6:457. doi: 10.3389/fmicb.2015.00457 (PMC4437033; doi:10.3389/fmicb.2015.00457)
Supplement: Supplementary file 1 [file DataSheet1.ZIP › S-Tables/S2.PDF]

Supplementary table s2: Reaction mixtures and PCR programs used for SiteFinding-PCR conducted using the MyCycler thermal cycler (Bio-Rad Laboratories, Hercules, CA)

Step	Reaction mixture	Amplification programs	No. of cycles
Site finding PCR	2 μl of 10×Taq DNA polymerase buffer, 2.5 mM each of dNTP, 2.5 U of Taq DNA polymerase, 10 pmol of SiteFinder1 (or SiteFinder 2) and 50 ng of template DNA, in a total volume of 20 μl	92 ℃ (2 min), 95 ℃ (1 min), 25 ℃ (1 min), ramped to 68 ℃ over 3 min, 68 ℃ (10 min)	1
Primary PCR	5 μl of mixture containing 50 pmol of SFP1, 10 pmol of -DF1 (or -UR1) and 1xTaq DNA polymerase buffer added to tubes from the site finding PCR	94 ℃ (1 min)<br>95 ℃ (10 s), 64 ℃ (6 min)<br>72 ℃ (10 min)	1<br>30<br>1
Secondary PCR	50 μl of mixture containing 1x Taq DNA polymerase buffer, 2.5 mM each of dNTP, 5 U of Taq polymerase, 0.2 mM of -DF2 (or -UR2) and SFP2, and 1 μl of 100x dilution of the products from the primary PCR as template. An additional PCR was performed using -DF3 (or -UR3) instead of -DF2 (or -UR2)	94 ℃ (1 min)<br>95 ℃ (10 s), 64 ℃ (6 min)<br>72 ℃ (10 min)	1<br>30<br>1
