# Supplementary material for: Heterologous expression of the Monilinia fructicola CYP51 (MfCYP51) gene in Pichia pastoris confirms the mode of action of the novel fungicide, SYP-Z048
Source: Front Microbiol. 2015 May 19;6:457. doi: 10.3389/fmicb.2015.00457 (PMC4437033; doi:10.3389/fmicb.2015.00457)
Supplement: Supplementary file 2 [file Image1.PDF]

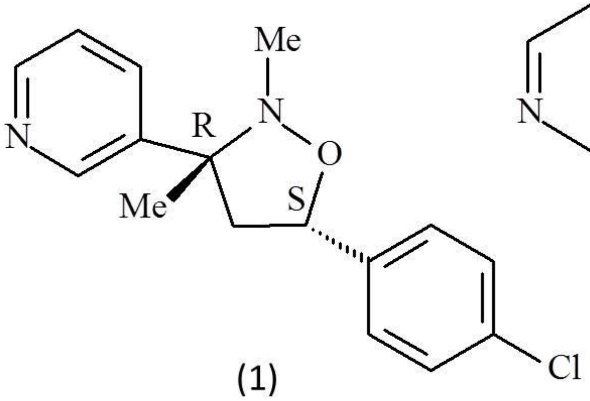

(1)

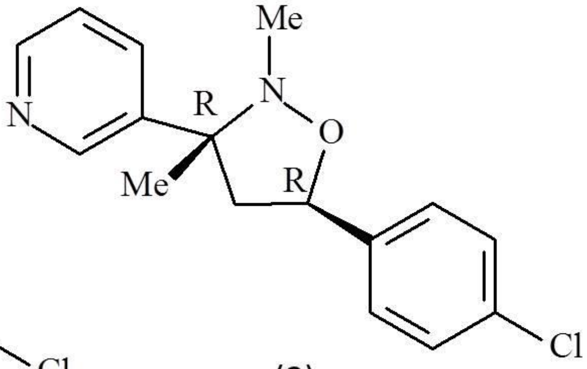

(2)

Supplementary figure s1. The fungicide SYP-Z048 used in this study consisted of a mixture of two stereoisomers: (1) 3-[(3R, 5S)-5-(4-chloro-phenyl)-2,3-dimethyl-3-isoxazolidinyl] pyridine and (2) its (3R, 5R)-isomer
