# Supplementary material for: Heterologous expression of the Monilinia fructicola CYP51 (MfCYP51) gene in Pichia pastoris confirms the mode of action of the novel fungicide, SYP-Z048
Source: Front Microbiol. 2015 May 19;6:457. doi: 10.3389/fmicb.2015.00457 (PMC4437033; doi:10.3389/fmicb.2015.00457)
Supplement: Supplementary file 1 [file DataSheet1.ZIP › S-Tables/S3.DOC]

Supplementary table s3: Nucleotide variations associated with the *ERG2*, *ERG24*, and *ERG27* genes of sensitive isolates and SYP-Z048-resistant mutants of *M. fructicola*

| Isolate |  |  |  | Variation at nucleotide position | | | | | | | | | | | | | |
| --- | --- | --- | --- | --- | --- | --- | --- | --- | --- | --- | --- | --- | --- | --- | --- | --- | --- |
|  |  | *ERG2* | *ERG24* | | | | | | | | | | |  | *ERG27* | | |
|  | Phenotype* |  | 505 | 604 | 674 | 696 | 709 | 835 | 853 | 1097 | 1171 | 1378 | 1462 |  | 828 | 866 | 1212 |
| MSB11 | S | Identical for all isolates | A | C | A | T | C | A | T | G | G | T | C |  | T | T | C |
| MPA18 | S |  | A | C | C | C | C | A | C | A | A | C | A |  | C | C | T |
| MFJ2 | S |  | G | T | C | C | T | G | T | A | A | T | A |  | C | C | T |
| A3081 | LR |  | A | C | A | A | C | A | T | G | G | T | C |  | T | T | C |
| B5013 | HR |  | A | C | A | A | C | A | T | G | G | T | C |  | T | T | C |
| B6012 | HR |  | A | C | A | A | C | A | T | G | G | T | C |  | T | T | C |
| B506 | HR |  | A | C | A | A | C | A | T | G | G | T | C |  | T | T | C |
| B511 | HR |  | A | C | A | A | C | A | T | G | G | T | C |  | T | T | C |

*S, LR and HR indicated that the isolates was sensitive, low resistant and highly resistant to SYP-Z048.
